# Identification and functional characterization of a potential l-Homoserine exporter in *Corynebacterium glutamicum*

**DOI:** 10.1016/j.engmic.2025.100240

**Published:** 2025-09-13

**Authors:** Xiaodi Liu, Xiangyu Zhu, Wenxin Jiang, Huanmin Du

**Affiliations:** a Department of Biomedical Engineering, City University of Hong Kong, 83 Tat Chee Avenue, Hong Kong, China; b Department of Mechanical and Energy Engineering, Southern University of Science and Technology, Shenzhen 518055, China; c Guangdong Key Laboratory of Fermentation and Enzyme Engineering, School of Biology and Biological Engineering, South China University of Technology, Guangzhou 510006, China

**Keywords:** Amino acid exporter, Cg0701, *Corynebacterium glutamicum*, L-Homoserine production

## Abstract

Exporter protein systems play a crucial role in the efficient production of valuable chemicals. However, the lack of active exporters significantly limits the application of industrial bio-based production, making the identification and utilization of novel exporters highly important. In this study, we discovered a novel l-Homoserine exporter, Cg0701, in *Corynebacterium glutamicum* through homology analysis. First, tolerance assays revealed that the *cg0701* overexpression strain (CgH-2) exhibited a 10.45% increase in cell growth compared to the control when cultivated with 30 g/L-Homoserine. Additionally, export assays demonstrated that the l-Homoserine export capacity of CgH-2 increased by approximately 30%. Furthermore, genomic overexpression of *cg0701* in an l-Homoserine-producing chassis also enhanced both tolerance and export activity. As a result, the recombinant strain CgH-11 produced 10.79 g/L-Homoserine in shake flask cultures and 48.72 g/L in a 5 L fermenter, representing improvements of 19.89% and 24.44%, respectively. In summary, our results indicate that Cg0701 is a novel l-Homoserine exporter in *C. glutamicum*, enriching our understanding of amino acid export systems and providing a valuable target for the construction of l-Homoserine microbial cell factories.

## Highlights

• A novel l-Homoserine exporter was identified in *C. glutamicum*.
• The exporter significantly enhances l-Homoserine tolerance and export capacity.
• The exporter greatly increases l-Homoserine production.

## Graphical abstract

**Figure:**
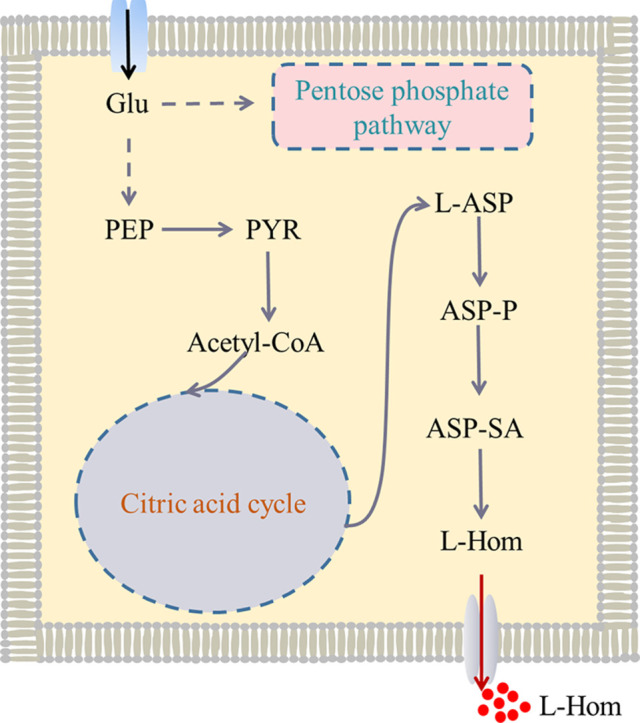

## 1 Introduction

L-Homoserine is a valuable non-proteinogenic amino acid that serves as an essential precursor for the biosynthesis of l-methionine and l-threonine [1,2]. It possesses a variety of physiological functions and has potential applications in food, medicine, cosmetics, agriculture, and animal feed [[3], [4], [5]]. Previous studies have highlighted l-Homoserine as a promising platform compound for the production of O-acetylhomoserine, 2,4-dihydroxybutyrate, 1,4-butanediol, and 1,3-propanediol [[6], [7], [8]]. In addition, l-Homoserine plays an important role in supporting chick growth and enhancing plant disease resistance [9,10]. Owing to its expanding applications and growing market demand, the production of l-Homoserine has attracted considerable attention. Currently, industrial production of l-Homoserine primarily relies on traditional chemical synthesis, which is associated with several significant drawbacks, including low yields, high costs of raw materials, and serious environmental pollution [11]. Therefore, it is necessary to develop a cost-effective, scalable, and environmentally friendly method to improve l-Homoserine production.

In recent years, the rapid advancement of systems biology and synthetic biology has increasingly highlighted the importance of constructing efficient microbial cell factories for l-Homoserine production [12]. *Escherichia coli* and *Corynebacterium glutamicum* are among the most widely used host strains for l-Homoserine biosynthesis, and their metabolic pathways—including glycolysis, the tricarboxylic acid (TCA) cycle, and the aspartate pathway—have been thoroughly elucidated [3,[13], [14], [15], [16]]. For instance, Li et al. (2023) engineered a *C. glutamicum* strain by enhancing the biosynthetic pathway, eliminating competitive pathways, and optimizing intracellular cofactor balance, achieving l-Homoserine titers of 16.8 g/L in shake flask cultures and 63.5 g/L in fed-batch fermentation [17]. Similarly, Vo et al. (2022) developed an efficient *E. coli* cell factory through systematic and rational metabolic engineering, resulting in a strain capable of producing 110.8 g/L-Homoserine with a productivity of 1.82 g/L/h in a 2 L fermenter [18]. Although significant progress has been made in microbial production of l-Homoserine, further improvements are necessary to enable large-scale industrial application. It is hypothesized that exporter systems may represent a key limiting factor in achieving higher production efficiencies.

Earlier studies have demonstrated that efficient exporters can maintain intracellular amino acid homeostasis, reduce feedback inhibition and cytotoxicity, and thereby enhance production capacity. As a result, exporters have been widely utilized in the industrial production of amino acids [19,20]. For example, Liu et al. (2015) identified a novel l-methionine exporter, YjeH, in *E. coli*, which significantly improved the export capacity of l-methionine and increased its titer by 70 % [21]. In another case, Zhang et al. (2020) characterized a new L‑serine transporter (SerE) in *C. glutamicum*, which greatly increased L‑serine tolerance and further enhanced its production in the SerE-overexpressing strain [22]. In our previous study, we found that RhtA acts as the major l-Homoserine exporter in *E. coli*, and overexpression of *rhtA* successfully increased both l-Homoserine resistance and production [11]. Therefore, enhancing efflux capacity is an effective strategy to improve l-Homoserine production performance. However, only a limited number of exporter proteins—such as RhtA and RhtB in *E. coli* and BrnFE in *C. glutamicum*—have been identified as l-Homoserine transporters [11,[23], [24], [25]]. Thus, the discovery of novel exporter proteins remains of great importance for further strain improvement.

*C. glutamicum* is a Gram-positive bacterium with GRAS (generally regarded as safe) status, originally isolated for the production of l-glutamate and now widely used as an ideal platform for the industrial production of various amino acids [26,27]. In *C. glutamicum*, approximately 750 membrane proteins—including 17 transporter families—have been identified as potential amino acid transporters [28]. These abundant transporter systems have significantly contributed to the advancement of the amino acid industry. Therefore, it is essential to intensify efforts to identify specific l-Homoserine exporters in *C. glutamicum*. In this study, homology analysis was first conducted to identify novel candidate l-Homoserine exporters. Subsequent analysis of l-Homoserine resistance and production confirmed that Cg0701 functions as an l-Homoserine exporter. Ultimately, a novel l-Homoserine exporter was successfully characterized in *C. glutamicum*, providing new insights into l-Homoserine transport systems and offering promising potential for enhanced l-Homoserine production.

## 2 Material and methods

### 2.1 Strains and growth conditions

All strains used in this study are listed in Table 1. The flowchart illustrating the construction of l-Homoserine exporter strains is shown in Fig. S1. *E. coli* DH5α was employed as the host for gene cloning and cultured at 37 °C in Luria-Bertani (LB) broth (5 *g*/L yeast extract, 10 *g*/L tryptone, and 10 *g*/L NaCl). *C. glutamicum* CgH-0 served as the parental strain and was grown in LBHIS medium (2.5 *g*/L yeast extract, 5 *g*/L tryptone, 18.5 *g*/L brain heart infusion powder, 5 *g*/L NaCl, and 91 *g*/L sorbitol) at 30 °C. When necessary, chloramphenicol was added to the medium at a final concentration of 30 μg/L for *E. coli* or 15 μg/L for *C. glutamicum*.

**Table 1:** Strains and plasmids used in this study.

Strains and plasmids	Characteristics	Source
*E.coli*		
*E. coli* DH5α	Host for plasmids construction	Invitrogen
*E. coli* MG1655	Host for genes cloning	Lab stock
*C. glutamicum*		
CgH-0	*C. glutamicum* ATCC13032	Lab stock
CgH-1	CgH-0, containing pXMJ19	This study
CgH-2	CgH-0, containing pXMJ-*cg0701*	This study
CgH-3	CgH-0, containing pXMJ-*cg294*1	This study
CgH-4	CgH-0, containing pXMJ-*rhtA* (*E. coli*)	This study
CgH-5	CgH-0, containing pXMJ-*rhtB* (*E. coli*)	This study
CgH-6	CgH-0, ∆*thrB*::P*sod*-*hom*, ∆*poxB*::P*sod*-*lysC*	This study
CgH-7	CgH-6, containing pXMJ19	This study
CgH-8	CgH-6, containing pXMJ19-*cg0701*	This study
CgH-9	CgH-6, △*cg0701*	This study
CgH-10	CgH-9, containing pXMJ19	This study
CgH-11	CgH-6, P*_cg0701_*:: P*_sod_*	This study
Plasmids		
pCRD206	*Kan^R^*, gene deletion plasmid	Lab stock
pCRD-△cg0701	pCRD206 derivate, deleting *cg0701*	This study
pCRD-P_sod_-cg0701	pCRD206 derivate, replacing native promoter of *cg0701* by the promoter P*_sod_*	This study
pXMJ19	*Cm^R^, C. glutamicum*-*E. coli* shuttle plasmid	Lab stock
pXMJ-cg0701	pXMJ19 derivate, inserting *cg0701* at MCS	This study
pXMJ-cg2941	pXMJ19 derivate, inserting *cg2941* at MCS	This study
pXMJ-rhtA	pXMJ19 derivate, inserting *rhtA* (*E. coli*) at MCS	This study
pXMJ-rhtB	pXMJ19 derivate, inserting *rhtB* (*E. coli*) at MCS	This study

### 2.2 Plasmids and strains construction

The plasmids used in this study are listed in Table 1. The *E. coli*–*C. glutamicum* shuttle vector pXMJ19 was used for gene expression, while pCRD206 was employed for gene deletion. The coding regions of the target genes were amplified from the genomic DNA of *C. glutamicum* ATCC 13,032 or *E. coli* MG1655 using specific primers (Table S1). PCR amplification was performed with Phusion High-Fidelity DNA Polymerase (NEB, USA). The purified PCR products were subcloned into the corresponding vectors by Gibson assembly to generate the recombinant plasmids [29]. The constructed expression plasmids were used for functional analysis, and the deletion plasmids were used for target gene knockout. Gene deletion, integration, and promoter substitution in *C. glutamicum* were achieved via two-step homologous recombination following the standard protocol described by Okibe et al. (2011) [30].

### 2.3 L-Homoserine tolerance analysis

For the l-Homoserine tolerance experiments, various concentrations of l-Homoserine were added to the cultures, and cell growth was used as the indicator. Briefly, single colonies of each strain were randomly picked from agar plates and cultivated overnight in LBHIS medium at 30 °C. The overnight cultures were washed three times and resuspended in phosphate-buffered saline (PBS, pH 7.4; 8 *g*/L NaCl, 0.2 *g*/L KH₂PO₄, 2.9 *g*/L Na₂HPO₄·12H₂O, and 0.2 *g*/L KCl). The resuspended cells were then inoculated into fresh LBHIS medium containing 0, 10, 20, or 30 *g*/L-Homoserine at an initial optical density at 600 nm (OD₆₀₀) of 0.1 in shake flasks. Cell growth (OD₆₀₀) was monitored at the indicated time points using a UV spectrophotometer (Xinmao Instrument, Shanghai, China).

### 2.4 L-Homoserine export assay

The export activity of l-Homoserine was evaluated according to a previously described standard method with minor modifications [11]. Briefly, the relevant strains were cultured overnight in LBHIS medium and then inoculated into fresh LBHIS medium at an initial OD₆₀₀ of 0.1. Cells were grown until the OD₆₀₀ reached 2.0, after which 40 *g*/L-Homoserine was added and incubation continued for an additional 4 h to allow intracellular accumulation of l-Homoserine. The cells were then harvested, washed three times with PBS buffer (pH 7.4), and resuspended in fresh LBHIS medium to initiate the l-Homoserine export process. The concentration of l-Homoserine in the supernatant was determined by high-performance liquid chromatography (HPLC). Export activity was assessed based on the amount of l-Homoserine exported into the supernatant.

### 2.5 Shake flask fermentation

For shake flask fermentation, recombinant strains were cultivated in 250 mL flasks containing 20 mL working volume. Single colonies of each strain were precultured overnight in LBHIS medium, then inoculated into seed medium at 5 % (v/v) and cultivated for 12 hours. The resulting seed cultures were transferred into fermentation medium at an initial OD₆₀₀ of 1.0 and further incubated for 72 hours for l-Homoserine production. The main components of the seed medium were: 30 *g*/L glucose, 20 *g*/L corn steep liquor, 5 *g*/L (NH₄)₂SO₄, 2 *g*/L urea, 0.5 *g*/L MgSO₄, and 0.5 *g*/L KH₂PO₄. The fermentation medium consisted of: 80 *g*/L glucose, 30 *g*/L corn steep liquor, 20 *g*/L Na₂S₂O₃, 1 *g*/L KH₂PO₄, 0.5 *g*/L MgSO₄, 0.5 *g*/L-methionine, 0.5 *g*/L-isoleucine, 0.01 *g*/L MnSO₄, 0.01 *g*/L FeSO₄, 1 mg/L vitamin B₁, 0.2 mg/L biotin, and 20 *g*/L CaCO₃. When necessary, 400 μM IPTG was added to the fermentation medium to induce plasmid expression.

### 2.6 Fed-batch fermentation in a 5 L fermenter

The relevant strains were first cultivated overnight in LBHIS medium and then transferred into seed medium at a 5 % (v/v) inoculation ratio. After 12 h of cultivation, the seed culture was inoculated into a fermenter (Baoxing Biotech Co., Shanghai, China) and cultivated for 72 h. The fermentation temperature was maintained at 30 °C, and the dissolved oxygen level was automatically regulated at 20 %. The pH was controlled at 7.0 by automatic addition of 25 % (v/v) NH₄OH. To maintain the glucose concentration at approximately 10–15 *g*/L, a 500 *g*/L glucose solution was continuously supplied as needed.

### 2.7 Analytical methods

Cell growth was monitored by measuring OD_600_ using a UV spectrophotometer (Xinmao Instrument, Shanghai, China). The concentration of l-Homoserine was determined by pre-column derivatization with O-phthalaldehyde (OPA), followed by high-performance liquid chromatography (HPLC) analysis. HPLC was performed using a Zorbax Eclipse-AAA column (4.6 mm × 150 mm, 5 μm; Agilent Technology, USA) at a column temperature of 40 °C, with detection at a wavelength of 210 nm [11].

## 3 Results

### 3.1 Identification of l-Homoserine export candidates in C. glutamicum

L-Homoserine is cytotoxic and is considered a limiting factor in its own production via fermentation [11,31]. To increase l-Homoserine productivity, enhancing its export from the intracellular to the extracellular environment is critical. However, to date, only one l-Homoserine exporter has been identified in *C. glutamicum* [25]. Therefore, we sought to identify novel l-Homoserine exporters in *C. glutamicum* and to investigate the impact of overexpressing these candidate exporters on l-Homoserine production in recombinant strains. Putative l-Homoserine exporter candidates in *C. glutamicum* were identified by performing BLAST searches (https://www.genome.jp/tools/blast/) using the known amino acid sequences of l-Homoserine exporters (RhtA and RhtB from *E. coli*) as queries. As summarized in Table 2, when the amino acid sequences of RhtA and RhtB were queried, Cg0701 and Cg2941 were identified as candidate l-Homoserine transporters, with extremely low E-values (3.0 × 10^–37^ and 3.0 × 10^–14^, respectively).

**Table 2:** Results from the homology search for l-Homoserine exporters in *C. glutamicum.*

L-Homoserine transporter	Identified protein from *C. glutamicum* and its annotation	Results in BLAST	
		Identity (%)	E-value
RhtA (*E. coli*)	Cg0701: Hypothetical protein, inner membrane transporter	39.62	3 × 10^–37^
RhtB (*E. coli*)	Cg2941: Threonine efflux protein	29.76	3 × 10^–14^

### 3.2 Functional characterization of l-Homoserine export candidates in C. glutamicum

**Fig. 1:**
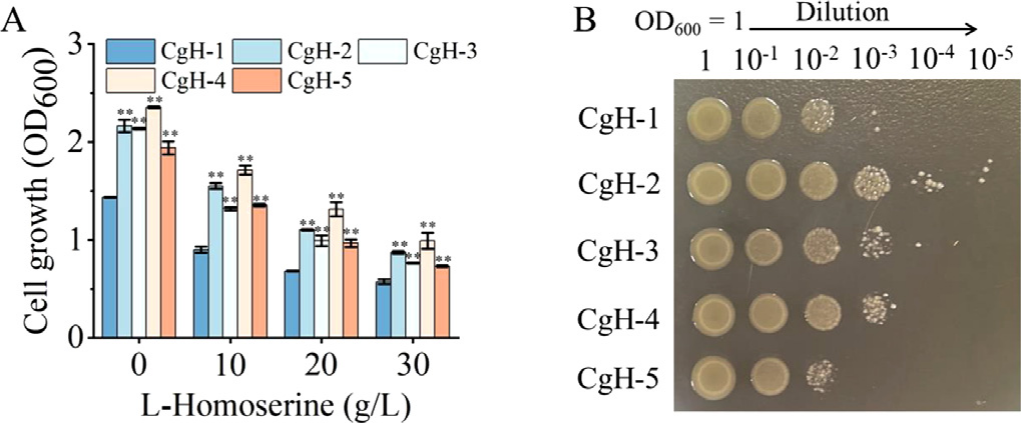
Functional characterization of the predicted l-Homoserine exporters in *C. glutamicum*. (A) The effect of varying concentrations of l-Homoserine on the growth of *C. glutamicum* strains overexpressing different candidate exporters was evaluated. Overnight cultures of *C. glutamicum* harboring the pXMJ19 empty vector or the predicted l-Homoserine exporter genes were inoculated into fresh LBHIS medium supplemented with different concentrations of l-Homoserine, at an initial OD_600_ of 0.1. Cultures were incubated at 30 °C for 6 h, and cell growth was monitored by measuring OD_600_. (B) The sensitivity of various strains carrying candidate l-Homoserine exporters to l-Homoserine was assessed on agar plates. The indicated strains were grown overnight in LBHIS medium, diluted to various concentrations, and then spotted onto agar plates containing 30 *g*/L-Homoserine. Data represent the mean values from three independent experiments, with standard deviations shown. ∗ *P* ≤ 0.05, ∗∗ *P* ≤ 0.01. CgH-1: CgH-0, containing pXMJ19; CgH-2: CgH-0, containing pXMJ-*cg0701*; CgH-3: CgH-0, containing pXMJ-*cg2941*; CgH-4: CgH-0, containing pXMJ-*rhtA* (*E. coli*); CgH-5: CgH-0, containing pXMJ-*rhtB* (*E. coli*).

To further characterize native l-Homoserine exporters, we explored the physiological significance of these candidates, including Cg0701 and Cg2941, in conferring l-Homoserine tolerance in *C. glutamicum*. As shown in Fig. S2, cell growth decreased as l-Homoserine concentrations increased. Specifically, when the concentration rose from 0 to 30 g/L, cell growth decreased by 60.42 %. However, strains overexpressing the predicted l-Homoserine exporters exhibited enhanced tolerance to l-Homoserine (Fig. 1). Notably, strains CgH-2 and CgH-3, which carried *cg0701* or *cg2941*, demonstrated significantly increased resistance to l-Homoserine. In particular, CgH-2 showed a 56.23 % increase in tolerance in the 30 g/L-Homoserine group compared to the non-exporter control strain CgH-1 after 6 h of cultivation (Fig. 1A). Similar results were observed in agar plate assays, with exporter-overexpressing strains displaying greater l-Homoserine tolerance (Fig. 1B).

### 3.3 Effect of Cg0701 on the export ability of l-Homoserine

**Fig. 2:**
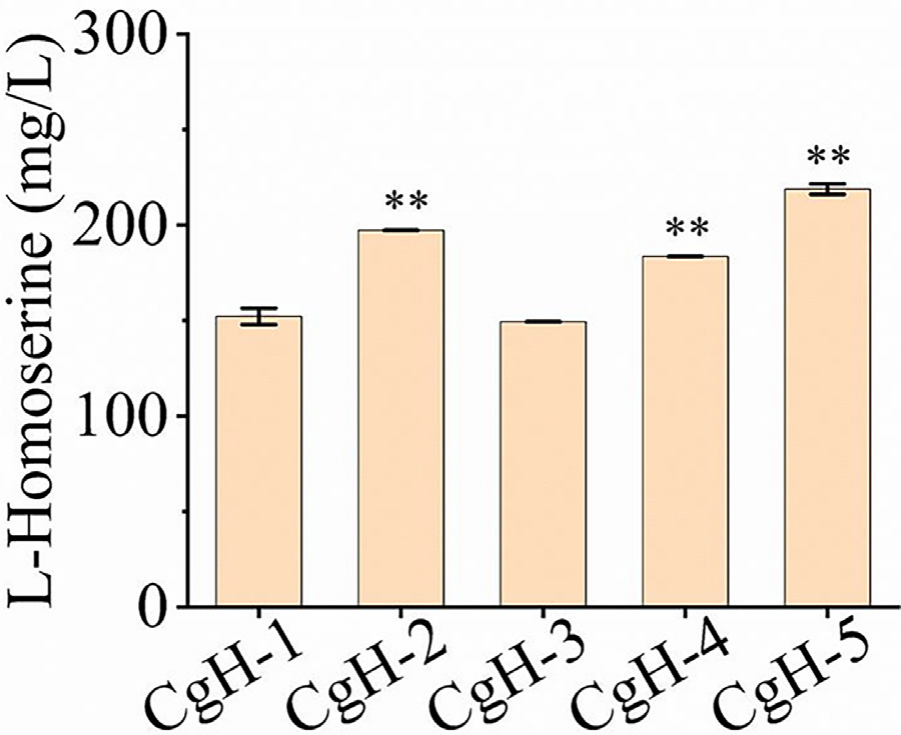
Determination of l-Homoserine export activity in different *C. glutamicum* strains. The indicated *C. glutamicum* strains were pre-cultured in LBHIS medium containing 40 *g*/L-Homoserine to allow intracellular accumulation of l-Homoserine. After cultivation, the cells were washed three times with PBS buffer (pH 7.4) and then resuspended in fresh LBHIS medium to initiate the l-Homoserine export process. The concentration of l-Homoserine in the supernatant was measured using HPLC. Data represent the mean values from three independent experiments, with standard deviations shown. ∗ *P* ≤ 0.05, ∗∗ *P* ≤ 0.01. CgH-1: CgH-0, containing pXMJ19; CgH-2: CgH-0, containing pXMJ-*cg0701*; CgH-3: CgH-0, containing pXMJ-*cg2941*; CgH-4: CgH-0, containing pXMJ-*rhtA* (*E. coli*); CgH-5: CgH-0, containing pXMJ-*rhtB* (*E. coli*).

Export efficiency is a key indicator of exporter functionality [11,32]. To better assess the l-Homoserine transport activity, we performed l-Homoserine export assays to compare the efflux capacity among five export-enhanced strains (CgH-1, CgH-2, CgH-3, CgH-4, and CgH-5). As shown in Fig. 2, all export-enhanced strains except CgH-3 exhibited increased l-Homoserine efflux to varying degrees. Notably, CgH-2 showed a 29.63 % increase in extracellular l-Homoserine concentration compared to the control strain CgH-1. To gain a better understanding of the potential transport mechanisms of Cg0701, its predicted structure was constructed by using AlphaFold Protein Structure Database and is depicted in Fig. S3. Based on these findings, we propose that Cg0701 is a novel l-Homoserine exporter in C. glutamicum.

### 3.4 Effect of exporter Cg0701 on l-Homoserine fermentation

**Fig. 3:**
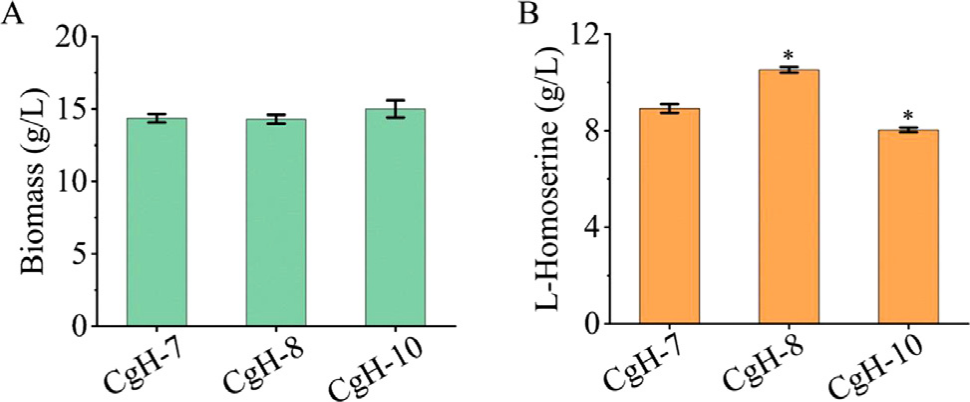
Fermentation profiles of the engineered strains were monitored throughout the fermentation process. The effects of *cg0701* on biomass (A) and l-Homoserine production (B) are presented. The various *cg0701*-engineered strains were cultivated in 250 mL flasks containing 20 mL of fermentation medium at 30 °C for 72 h. Data represent the mean values from three independent experiments, with standard deviations shown. ∗ *P* ≤ 0.05, ∗∗ *P* ≤ 0.01. CgH-7: CgH-6, containing pXMJ19; CgH-8: CgH-6, containing pXMJ19-*cg0701*; CgH-10: CgH-9, containing pXMJ19.

Efficient exporters play a vital role in enhancing l-Homoserine production [3,11]. Accordingly, we conducted shake flask fermentations to evaluate the effect of Cg0701 on l-Homoserine production. Three exporter-containing strains (CgH-7, CgH-8, and CgH-10) were constructed. The fermentation profiles indicated no significant differences in cell growth among the three l-Homoserine-producing strains (Fig. 3A). As shown in Fig. 3B, l-Homoserine production by CgH-8 (overexpressing *cg0701*) reached 10.52 g/L, approximately 20 % higher than that of the control strain CgH-7 (8.92 g/L), whereas the yield of the deletion strain CgH-10 was slightly reduced.

### 3.5 Genome-level overexpression of *cg0701* enhances l-Homoserine production

**Fig. 4:**
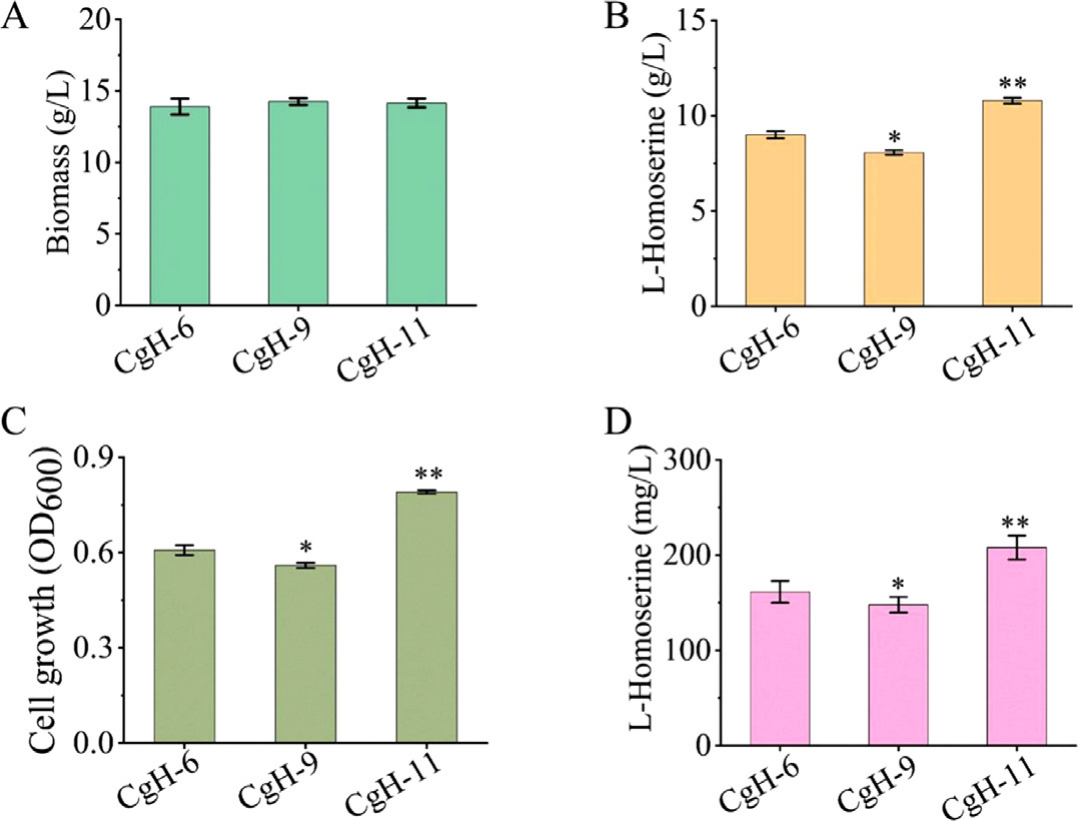
Assessing the genome-level impact of *cg0701* on l-Homoserine production. (A) Effect of *cg0701* on cell growth in engineered *C. glutamicum* strains. (B) Effect of *cg0701* on l-Homoserine production in recombinant strains. Recombinant strains harboring *cg0701* were cultivated in 250 mL flasks with a 20 mL working volume at 30 °C for 72 h. l-Homoserine production was measured at the end of the incubation period. (C) Effect of *cg0701* on l-Homoserine tolerance in recombinant strains. Overnight cultures of the indicated recombinant strains were inoculated into fresh LBHIS medium supplemented with 30 *g*/L-Homoserine at an initial OD_600_ of 0.1 and incubated at 30 °C for 6 h. Cell growth was monitored by measuring OD_600_. (D) Effect of *cg0701* on l-Homoserine export activity in engineered *C. glutamicum* strains. The indicated strains were pre-cultivated in LBHIS medium containing 40 *g*/L-Homoserine to promote intracellular accumulation. After washing three times with PBS buffer (pH 7.4), cells were resuspended in fresh LBHIS medium to initiate the l-Homoserine export process. l-Homoserine concentrations in the supernatant were determined by HPLC. Data represent the mean values from three independent experiments, with standard deviations shown. ∗ *P* ≤ 0.05, ∗∗ *P* ≤ 0.01. CgH-6: CgH-0, ∆*thrB*::P*sod*-*hom*, ∆*poxB*::P*sod*-*lysC*; CgH-9: CgH-6, △*cg0701*; CgH-11: CgH-6, P*_cg0701_*:: P*_sod_*.

Previous studies have shown that overexpression of key genes via recombinant plasmids can improve the production of biochemical [[33], [34], [35]]. However, plasmid-based expression systems often require antibiotics to maintain plasmid stability during fermentation, which can impose a metabolic burden and reduce overall biomass. Additionally, costly inducers such as IPTG may be needed to control gene expression. In this study, we replaced the native promoter of *cg0701* with the strong P*_sod_* promoter to enhance its expression, generating strain CgH-11. To evaluate the effect of *cg0701* overexpression at the genome-level, three strains (CgH-6, CgH-9, and CgH-11) were analyzed. As shown in Fig. 4A, there were no significant differences in final biomass among the three strains. l-Homoserine production by CgH-11 reached 10.79 g/L, representing a 19.89 % increase compared to the control strain CgH-6 (9.00 g/L) (Fig. 4B). In contrast, the *cg0701*-deficient strain CgH-9 accumulated 8.87 g/L-Homoserine after 72 h of fermentation, comparable to the control. Furthermore, compared to CgH-6, the *cg0701*-overexpressing strain CgH-11 showed significant improvements in l-Homoserine tolerance (10.45 % increase) and export capacity (28.89 % increase) (Fig. 4C and 4D). Conversely, the *cg0701*-deficient strain CgH-9 showed decreases of 8.96 % and 8.26 % in tolerance and exporter activity, respectively. These results demonstrate that Cg0701 is a novel l-Homoserine exporter that can increase l-Homoserine production by enhancing both tolerance and efflux capacity.

### 3.6 Fed-batch fermentation for l-Homoserine production

**Fig. 5:**
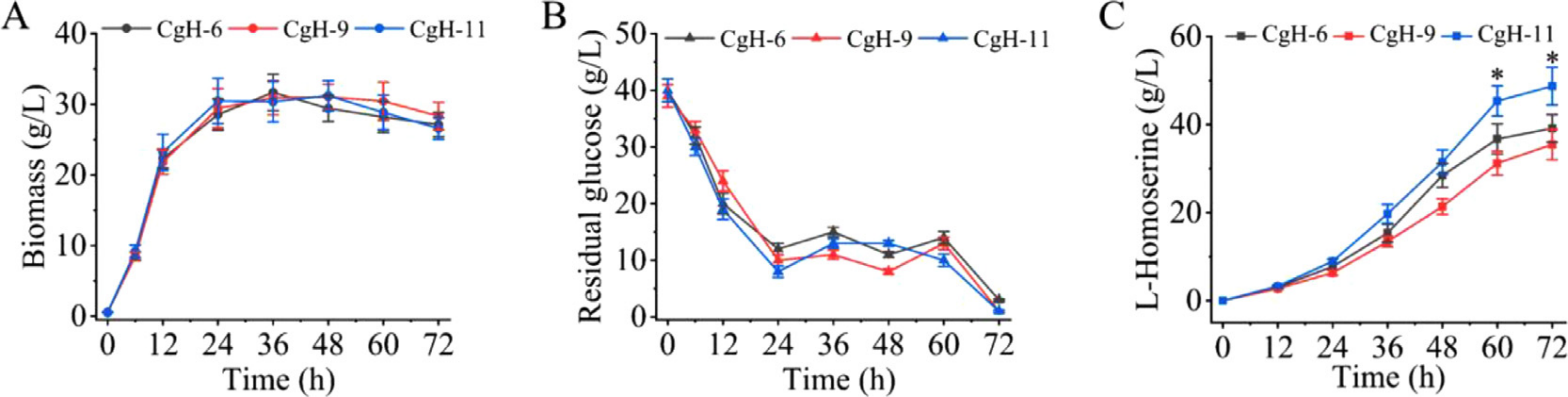
Fed-batch fermentation profiles of different *cg0701*-engineered strains in a 5 L fermenter. Time-course profiles of cell growth (A), residual glucose (B) and l-Homoserine production (C) were monitored during fed-batch cultivation of the various *cg0701*-engineered strains in a 5 L fermenter. Data represent the mean values from three independent experiments, with standard deviations shown. CgH-6: CgH-0, ∆*thrB*::P*sod*-*hom*, ∆*poxB*::P*sod*-*lysC*; CgH-9: CgH-6, △*cg0701*; CgH-11: CgH-6, P*_cg0701_*:: P*_sod_*.

To comprehensively evaluate the fermentation characteristics of the novel exporter Cg0701, fed-batch cultures of strains CgH-6, CgH-9, and CgH-11 were carried out in 5 L fermenters with working volumes of 3 L. The dynamics of cell growth (Fig. 5A) and glucose consumption (Fig. 5B) were continuously monitored during the entire fed-batch cultivation. Cell growth gradually increased to its peak and then declined slightly, and the final biomass of CgH-11 reached 26.58 g/L (Fig. 5A). Concurrently, l-Homoserine production in CgH-11 reached 48.72 g/L, representing a 24.44 % increase compared to the control strain CgH-6 (39.15 g/L) (Fig. 5C).

## 4 Discussion

With the rapid advancement of metabolic engineering and synthetic biology, microorganisms have emerged as promising platforms for the production of various chemicals [[36], [37], [38]]. However, product toxicity and metabolic flux control remain significant challenges, highlighting the crucial role of exporter systems in improving yields [[39], [40], [41]]. In this study, we identified Cg0701 as a novel l-Homoserine exporter in *C. glutamicum*, supported by several lines of evidence: (i) deletion of *cg0701* increased cellular sensitivity to l-Homoserine, whereas overexpression of *cg0701* enhanced cellular resistance; (ii) the *cg0701*-overexpressing strain exhibited significantly higher l-Homoserine export activity compared to the control; and (iii) overexpression of *cg0701* in an l-Homoserine-producing strain substantially increased l-Homoserine production, demonstrating its functional relevance from an application perspective.

Previous studies have demonstrated the pivotal roles of transporter systems in amino acid accumulation. For instance, Malla et al. (2022) identified an efficient l-lysine exporter, MglE, through functional metagenomic selection. Expression of *mglE* increased l-lysine tolerance in *E. coli* by 40 % and improved l-lysine yield in *C. glutamicum* by 7.8 % [42]. Similarly, Ding et al. (2023) identified and characterized the l-Homoserine transporter RhtA, and, through in vivo continuous directed evolution and growth-coupled selection, obtained beneficial mutants that increased l-Homoserine tolerance and yield by more than 5-fold and 3-fold, respectively [11]. In this study, we successfully identified and characterized a novel l-Homoserine exporter, Cg0701, which significantly enhanced both l-Homoserine resistance and transport capacity in *C. glutamicum*. Furthermore, genome-level overexpression of *cg0701* in the l-Homoserine-producing chassis strain CgH-6 led to a marked improvement in production, with a 24.44 % increase compared to the control strain.

In conclusion, our study has identified Cg0701 as a novel l-Homoserine exporter, advancing our understanding of amino acid export systems and offering a promising target for engineering efficient l-Homoserine-producing microbial strains.

## Data Availability Statement

All data generated during this study are included in this published article and its supplementary information files. The original data and resources are available from the corresponding author on reasonable request.

## CRediT authorship contribution statement

**Xiaodi Liu:** Writing – original draft, Conceptualization. **Xiangyu Zhu:** Methodology, Investigation, Data curation. **Wenxin Jiang:** Validation, Methodology. **Huanmin Du:** Writing – review & editing, Supervision, Conceptualization.

## Declaration of Competing Interest

The authors declare that they have no known competing financial interests or personal relationships that could have appeared to influence the work reported in this paper.

## Appendix Supplementary materials

Image, application 1
