# Supplementary material for: Identification and functional characterization of a potential l-Homoserine exporter in Corynebacterium glutamicum
Source: Eng Microbiol. 2025 Sep 13;5(4):100240. doi: 10.1016/j.engmic.2025.100240 (PMC12967821; doi:10.1016/j.engmic.2025.100240)
Supplement: Supplementary file 1 [file mmc1.docx]

Identification and functional characterization of a potential L-Homoserine exporter in *Corynebacterium glutamicum*

Xiaodi Liu^1, 2^, Xiangyu Zhu^3^, Wenxin Jiang^1^, Huanmin Du^1*^

1. Department of Biomedical Engineering, City University of Hong Kong, 83 Tat Chee Avenue, Hong Kong, China

2. Department of Mechanical and Energy Engineering, Southern University of Science and Technology, Shenzhen, 518055, China.

3. Guangdong Key Laboratory of Fermentation and Enzyme Engineering, School of Biology and Biological Engineering, South China University of Technology, Guangzhou 510006, China

*To whom correspondence should be addressed:

Huanmin Du; E-mail: [huanmidu@cityu.edu.hk](mailto:huanmidu@cityu.edu.hk)


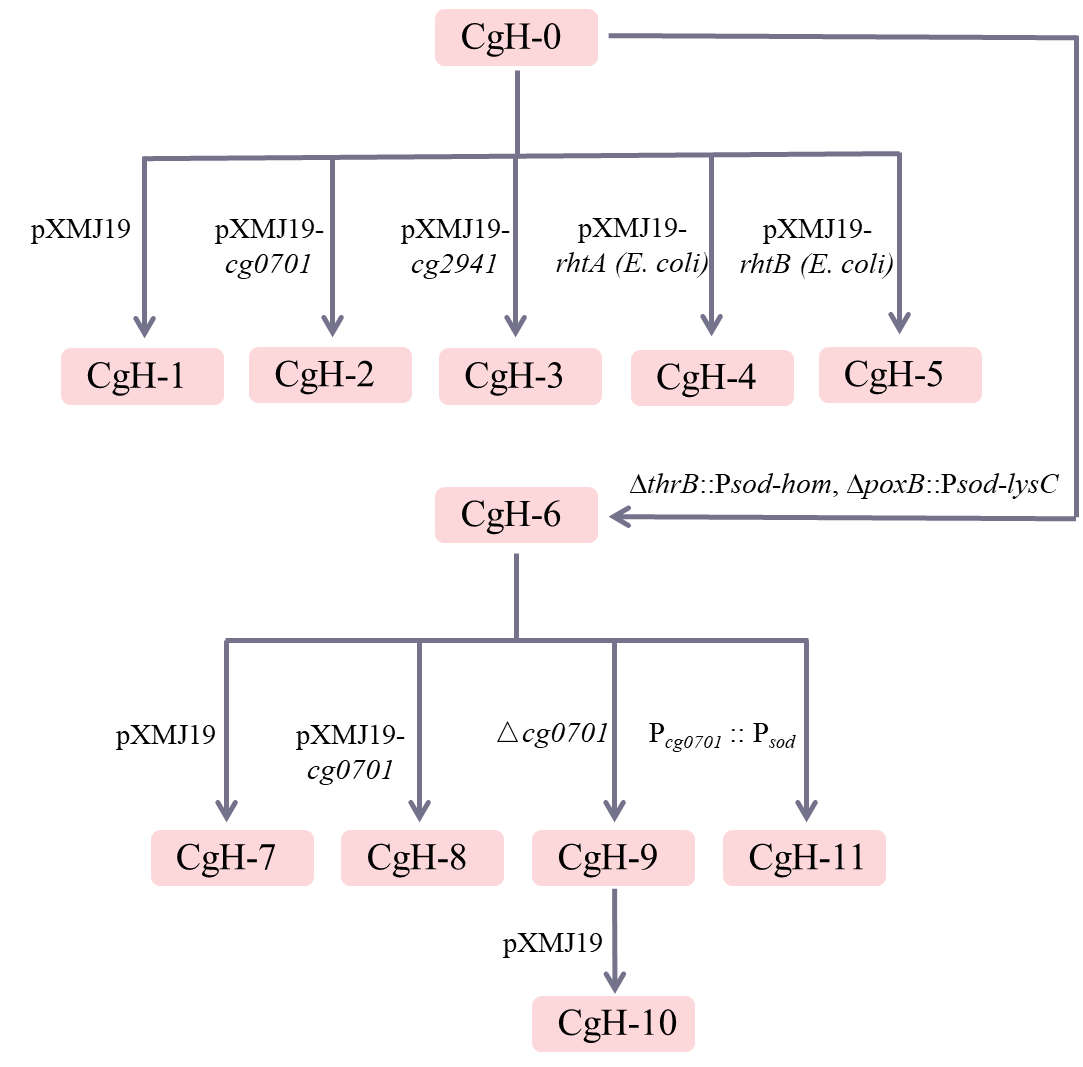


Fig. S1 Flowchart illustrating the construction of L-Homoserine exporter strains in *C. glutamicum*.


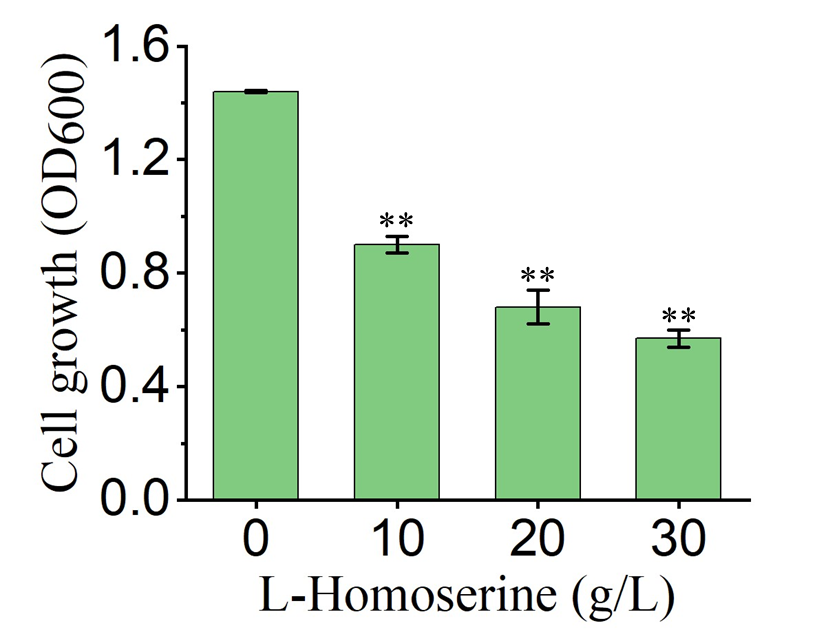


Fig. S2 Effect of different L-Homoserine concentrations on the growth of CgH-0. Data represent the mean values from three independent experiments, with standard deviations shown. ∗ P ≤ 0.05, ∗∗ P ≤ 0.01.


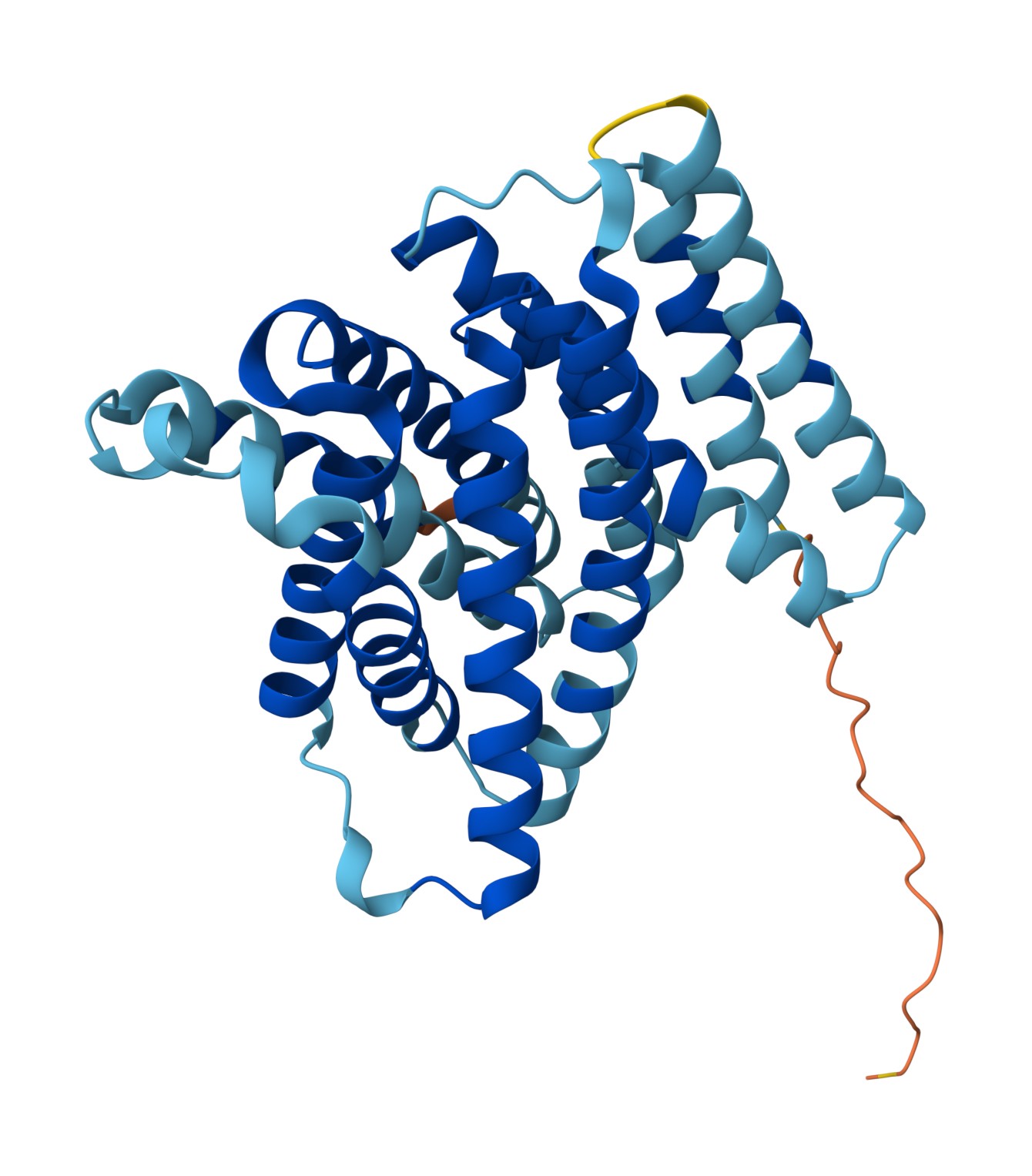
Fig. S3 The predicted structure of Cg0701 exporter.

Table S1 Primers used in this study

| Name | Sequence (5’-3’) |
| --- | --- |
| Primers for the pCRD206 | |
| pCRD206-F | ggatccccgggtaccgagctcgaattc |
| pCRD206-R | tctagagtcgacctgcaggcatgcaagct |
| pCRD-cg0701-1F | cgaattcgagctcggtacccggggatcctggggttgccgggcagcggctggatgtgca |
| pCRD-cg0701-1R | gatcaagcaggatccagccgaagcctgcgatgaacagcct |
| pCRD-cg0701-2F | aggctgttcatcgcaggcttcggctggatcctgcttgatc |
| pCRD-cg0701-2R | aagcttgcatgcctgcaggtcgactctagagggcaagcagggtggccttggcgtgatc |
| pCRD-Psod-cg0701-1F | ttacgaattcgagctcggtacccggggatccactctactagacgagcctccaaataag |
| pCRD-Psod-cg0701-1R | aattggcagctaagtagggtgcccttgattattgccaaag |
| pCRD-Psod-cg0701-2F | ctttggcaataatcaagggcaccctacttagctgccaatt |
| pCRD-Psod-cg0701-2R | gcagcggactgtttattcattgggtaaaaaatcctttcgt |
| pCRD-Psod-cg0701-3F | acgaaaggattttttacccaatgaataaacagtccgctgc |
| pCRD-Psod-cg0701-3R | caagcttgcatgcctgcaggtcgactctagaaagcttggagcagattctgcatggtc |
| pXMJ19-F | ggatccccgggtaccgagctcgaattcagctt |
| pXMJ19-R | tctagagtcgacctgcaggcatgcaagcttaatt |
| pXMJ-Cg0701-F | taagcttgcatgcctgcaggtcgactctagaatgaataaacagtccgctgcagtgttga |
| pXMJ-Cg0701-R | aagctgaattcgagctcggtacccggggatccttaactaggtgtgtgtactcgcctct |
| pXMJ-Cg2941-F | taagcttgcatgcctgcaggtcgactctagagtggacgcagcatcatgggtcgcattcg |
| pXMJ-Cg2941-R | aagctgaattcgagctcggtacccggggatccctaagtgataagcccataagcagtat |
| pXMJ-rhtA-F | aagcttgcatgcctgcaggtcgactctagaatgcctggttcattacgtaaaatgccggt |
| pXMJ-rhtA-R | aagctgaattcgagctcggtacccggggatccttaattaatgtctaattcttttatt |
| pXMJ-rhtB-F | aagcttgcatgcctgcaggtcgactctagaatgaccttagaatggtggtttgcctacct |
| pXMJ-rhtB-R | aagctgaattcgagctcggtacccggggatcctcacgcatgcctcgccgatgctaacag |
